# Optimization Strategies for Mass Spectrometry-Based Untargeted Metabolomics Analysis of Small Polar Molecules in Human Plasma

**DOI:** 10.3390/metabo13080923

**Published:** 2023-08-07

**Authors:** Michał Kaczmarek, Nanyun Zhang, Ludmila Buzhansky, Sharon Gilead, Ehud Gazit

**Affiliations:** 1 Division of Metabolite Medicine, Blavatnik Center for Drug Discovery, Tel Aviv University, Tel Aviv 69978, Israel; nanyun@tauex.tau.ac.il (N.Z.); ludmilabu@tauex.tau.ac.il (L.B.); sharong@tauex.tau.ac.il (S.G.); ehudga@tauex.tau.ac.il (E.G.); 2 The Shmunis School of Biomedicine and Cancer Research, Tel Aviv University, Tel Aviv 69978, Israel

**Keywords:** untargeted metabolomics, method optimization, liquid chromatography, mass spectrometry, plasma, LC-MS

## Abstract

The untargeted approach to mass spectrometry-based metabolomics has a wide potential to investigate health and disease states, identify new biomarkers for diseases, and elucidate metabolic pathways. All this holds great promise for many applications in biological and chemical research. However, the complexity of instrumental parameters on advanced hybrid mass spectrometers can make the optimization of the analytical method immensely challenging. Here, we report a strategy to optimize the selected settings of a hydrophilic interaction liquid chromatography-tandem mass spectrometry method for untargeted metabolomics studies of human plasma, as a sample matrix. Specifically, we evaluated the effects of the reconstitution solvent in the sample preparation procedure, the injection volume employed, and different mass spectrometry-related operating parameters including mass range, the number of data-dependent fragmentation scans, collision energy mode, duration of dynamic exclusion time, and mass resolution settings on the metabolomics data quality and output. This study highlights key instrumental variables influencing the detection of metabolites along with suggested settings for the IQ-X tribrid system and proposes a new methodological framework to ensure increased metabolome coverage.

## 1. Introduction

Metabolomics is one of the latest ‘omics’ approaches that aims to measure a wide range of metabolites in biological specimens. Unlike genes and proteins, metabolites, the small molecules that are transformed during metabolism, provide a functional readout of cellular biochemistry best representing the molecular phenotype. Therefore, comparison of the metabolic profiles of different samples (e.g., control versus disease, drug versus placebo, etc.) has become a common approach for monitoring and discovering novel biomarkers of normal and abnormal states, shaping the understanding of human physiology and holding great promise for personalized medicine [1,2].

Metabolomics approaches can be divided into targeted and untargeted. Targeted metabolomics is a quantitative measurement examining a relatively limited and definite number of metabolites. This approach is employed in studies investigating specific metabolic pathways or validating biomarkers identified in untargeted metabolomics [3]. Untargeted metabolomics, in contrast, aims to maximize the number of metabolites detected, thus involving global profiling of the holistic collection of metabolites (metabolome), and it is typically employed in hypothesis-generating studies such as biomarker discovery. Untargeted metabolomics involves the measurement of as many metabolites as possible, without any prior knowledge or targeting of specific compounds. It often provides more information as compared to the targeted metabolomics approach [4,5,6].

Given the diversity of chemical classes and physical properties that define metabolites, as well as the dynamic range of metabolite concentrations across large orders of magnitude, numerous analytical methods have been applied in metabolomics research. Among these, mass spectrometry (MS) is the predominant analytical technique employed in metabolomic profiling [7]. Current MS analyzers, such as Orbitrap, Fourier-transform ion cyclotron resonance, and time of flight mass spectrometers, are capable of a very high mass resolution and provide high mass accuracy. Such systems can be used to distinguish between molecules with the same nominal mass and determine plausible elemental compositions by accurate measurement of mass-to-charge ratio (*m*/*z*) and examination of isotope patterns. With recent advancements in MS technology, new commercially available instrument designs combining different performance characteristics offered by various types of analyzers have been developed. For instance, hybrid instruments are capable of the spatiotemporal separation of ion isolation and advanced fragmentation, offering additional structural information [8]. Coupling such instruments with high-resolution chromatography technologies, for example, ultra-performance liquid chromatography (UPLC), creates a powerful combination capable of resolving hundreds to thousands of individual metabolites from a single analysis [9].

Chromatography columns are an essential component of UPLC systems, and their type is an important factor influencing the performance of the analysis. In metabolomics, the most widely adopted LC approaches are reversed-phase liquid chromatography (RPLC) and hydrophilic interaction liquid chromatography (HILIC) [2]. RPLC is employed in most untargeted studies, as it generates reproducible data for a large set of non- to moderately polar metabolites. Recently, however, with the introduction of reproducible and robust stationary phases, HILIC has gained popularity in metabolomics research [10,11,12]. HILIC provides complementary selectivity to RPLC, retaining moderately to highly polar analytes [13], and coupled with MS has been frequently used for studies of metabolites in human plasma [14], urine [15], bacterial extracts [16], and many other biological samples [10]. Thus, it is not surprising that over the last decade, liquid chromatography coupled with mass spectrometry (LC-MS) has become the most common approach for MS-based metabolomics owing to its wide metabolite coverage, speed, high sensitivity, and ease of sample preparation [2,9].

Biofluids are predominantly aqueous and thus are composed of numerous water-soluble analytes [10]. Plasma, for instance, contains a number of hydrophilic primary metabolites that are predominantly polar [17]. HILIC is therefore an appropriate method of separation of blood plasma metabolites, as it covers polar and ionic compounds that are not retained in RPLC, where they elute in the solvent front and may be subjected to ion suppression [11,17,18]. Moreover, HILIC chromatography conditions have been found to yield higher sensitivity and more favorable ionization conditions of small organic molecules as compared to RPLC [19].

Here, we set out to expand the coverage of the detected metabolome by optimizing instrumental conditions of an advanced tribrid mass spectrometer Orbitrap IQ-X system, employing previously used chromatographic conditions [20]. Specifically, the present study aimed to optimize the composition of the reconstitution solvent used in the sample preparation procedure, the injection volume, and the mass spectrometry parameters of the HILIC-LC-MS method for improved detection of polar metabolites in untargeted metabolomics studies of human biospecimens.

## 2. Materials and Methods

### 2.1. Reagents and Materials

Acetonitrile and methanol (Lichrosolv^®^ grade for LC-MS analysis) were obtained from Supleco (Merck, Darmstadt, Germany). Water (18.2 MΩ·cm) was purified in a Milli-Q device, Millipore Purification System (Merck, Darmstadt, Germany). Ammonium formate and formic acid (LiChropur™ LC−MS grade) were purchased from Sigma-Aldrich (St. Louis, MO, USA). Human plasma was obtained from Sigma-Aldrich (P9523, St. Louis, MO, USA).

### 2.2. Plasma Sample Preparation

Frozen plasma samples were thawed at room temperature and protein precipitation was performed with 800 μL of ice-cold MeOH added to 200 μL of each plasma replicate (n = 16, which were later divided into four different groups for reconstitution). After shaking for 30 s in a Mini Mixer (MIX-30S, MIULab, Hangzhou, China) and one-hour incubation at −20 °C, samples were centrifuged at 16,000× *g* at 4 °C for 15 min in a 5430 R centrifuge (Eppendorf, Hamburg, Germany). The supernatants were transferred to a 1.5 microcentrifuge tube and evaporated to dryness in a Concentrator plus (Eppendorf, Hamburg, Germany) at 30 °C. Dry extracts were reconstituted in 200 μL of the appropriate mixture of methanol/water (6:4, 7:3, 8:2, 9:1, *v*/*v*, 4 replicates per group), sonicated for 1 min in an ice-filled ultrasonic bath system (MRC Scientific Instruments, Holon, Israel), and centrifuged at 16,000× *g* at 4 °C for 15 min. The supernatants were then transferred into a Nanosep MF centrifugal device with Bio-Inert 0.2 μm membrane (Pall, Port Washington, NY, United States), filtered, and transferred into injection UPLC vials with 0.2 mL inserts for analysis.

### 2.3. HILIC Chromatography

Chromatography was performed on an Accucore-150-Amide-HILIC column (100 × 2.1 mm, 2.6 μm, Thermo Fisher Scientific, MA, USA) using a Thermo Scientific Vanquish Flex UHPLC System (Thermo Fisher Scientific, Waltham, MA, USA). The chromatography method employed was modified from Southam’s method [20]. Briefly, the column oven temperature was maintained constant at 35 °C. Separation was performed under gradient elution using a binary mobile phase system consisting of solvent A: acetonitrile/methanol/water (90:5:5, *v*/*v*/*v*) and solvent B: water/acetonitrile (50:50, *v*/*v*). Both solvents contained 10 mM ammonium formate and 0.1% formic acid. The gradient elution program, presented in Table 1, started with a 1-min isocratic step at 99% A, then rising to 25% B linearly over 2 min, to 50% B over the next 3 min, and finally to 95% B over 3 min. This composition was maintained until the 10 min mark, after which it decreased to a starting composition of 5% B over 0.5 min, equilibrating the column for the following run for the next 4.5 min. The flow rate was 0.5 mL/min. The injection system was subjected to two washing cycles with 90:10 water/methanol containing 0.1% formic acid before and after the injection. To minimize the batch effect while assessing the parameters, samples were randomized by using a home-written R script. We have divided samples into five analytical blocks; each block consisted of samples from all investigated groups with the parameter assessed ordered randomly.

### 2.4. Detection and Electrospray Ionization

MS detection was carried out on an Orbitrap IQ-X Tribrid Mass Spectrometer (Thermo Fisher Scientific, Waltham, MA, USA). Electrospray ionization, achieved using an EASY-Max NG ion source (OPTON-30157), was operating in a positive ion mode. The ion spray voltage was set at 3500 V. Sheath, auxiliary, and sweep gas parameters were 60, 15, and 2 (arbitrary units), respectively. The ion transfer tube temperature was set at 350 °C and the vaporizer temperature at 400 °C.

### 2.5. Orbitrap-Based Mass Spectrometry Experiment

**Scheme 1:**
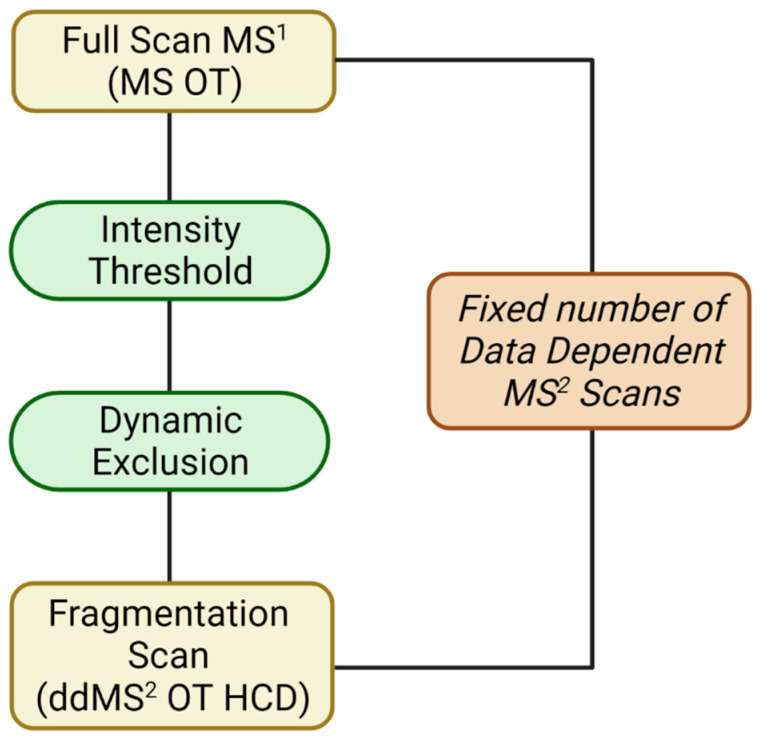
Block diagram of mass spectrometry experiment.

The employed mass spectrometry experiment is presented in Scheme 1. The experiment started with a full MS scan performed on the Orbitrap (MS OT). In this scan, the RF Lens was set to 35% and the maximum injection time was 50 ms. To improve the mass accuracy, real-time internal mass calibration of the Orbitrap analyzer was employed by generating calibrant ions of fluoranthene with an EASY-IC™ ion source. The intensity threshold, the minimum signal intensity required to trigger a data-dependent scan, was set to 2.0 e4. In the presented MS experiment, the user-selected number of most abundant ions within each full scan was subjected to fragmentation (Orbitrap-measured Data Dependent Fragmentation Scans activated by higher energy collisional dissociation—ddMS^2^ OT HCD). To register more unique ddMS^2^ OT HCD within the run, dynamic exclusion was employed. In the exclusion procedure, the *m*/*z* of the most intensive ions selected for fragmentation were added to a temporary ‘exclusion’ list for a desired period of time over the following cycles so that the same ions would not be fragmented repeatedly.

### 2.6. Computational Analysis of MS Data

All obtained UPLC-MS/MS .RAW files were processed using Compound Discoverer™ 3.3.1.111 (Thermo Fisher Scientific, Waltham, MA, USA). Untargeted metabolomics workflow (Supplementary Material Figure S1) was employed to perform spectra selection, retention time (RT) alignment, detection and grouping of features (chromatographic peaks with the same *m*/*z* × RT dimensions), gap filling, and the tentative compound identification with an online mass spectral database—mzCloud. All detected features were filtered by the group’s coefficient of variation (CV) of integrated peak areas, as it is a common measure of the variability of data points, calculated as the ratio of standard deviation to mean value. Only features with a CV below 30% were considered, as a CV value above this threshold would imply highly dispersed data [21].

When peak shape was considered, the performance was assessed visually in the FreeStyle™ 1.8 SP2 (Thermo Fisher Scientific, Waltham, MA, USA). The visual assessment took into account the similarity of a peak to the Gaussian shape and the presence, absence, or severity of peak tailing, peak fronting, and peak splitting. This assessment was performed on a set of commonly reported plasma metabolites, i.e., betaine, carnitine, creatine, acetylcholine, citrulline, trigonelline, tryptophan, ergothioneine, asparagine, hydroxyproline, lysine, glutamine, ornithine, serine, threonine, valine, alfoscerate, β-alanine, and acylcarnitine. The same set of compounds was also used to assess the linearity of the instrumental response employing the linear regression model. Statistical analyses of the results, including principal component analysis (PCA), comparison of feature intensities, and heatmap analysis, were performed using home-written R scripts. Heatmap analysis uses an agglomerative approach (hierarchical cluster analysis) to find the similarities between samples and compounds. Initially, the algorithm assigns each compound to its own singleton cluster. The analysis then proceeds iteratively, at each stage joining the two most similar clusters into a new cluster, continuing until there is one overall cluster represented by a dendrogram.

## 3. Results and Discussion

We investigated the impact of the composition of the reconstitution solvent used during the sample preparation procedure, the injection volume in the UPLC, and five different mass spectrometry parameters on the data output. For each of the parameters, individual assessment criteria were employed to identify the optimal value. The examined retention time range (main elution range) was between the beginning of a run and the column equilibration phase, from 0.5 to 10 min. Each parameter testing was performed by incorporating a base (“default”) method so that only one factor was changed each time. The base parameters are listed in Table 2.

To ensure that the variance in metabolites profile originates from different instrumental settings, rather than differences in sample preparation, after the assessment of the effect of the reconstitution solvent, the four replicates of 80:20 (*v*/*v*) acetonitrile: water extracts were combined and divided into four homogenous aliquots that were used in subsequent assessments. For each assessment, five technical replicates per investigated group were performed.

### 3.1. Reconstitution Solvent

A commonly reported issue in the application of HILIC chromatography is the distortion of peak shapes originating from a mismatch between the reconstitution solvent and the mobile phase [22]. An improper solvent in the reconstitution step can negatively affect the peak shape of metabolites and influence their retention times, thus affecting the method’s sensitivity and the total number of peaks detected.

In this study, we compared the solvents composed of acetonitrile and water at 60:40, 70:30, 80:20, and 90:10 (*v*/*v*). Assessment of this parameter was based on:

- Total number of features found in MS OT.
- Distribution of elution features over the retention time.
- Number of matches with mzCloud Best Match score >80%.
- Visual evaluation of the peak shapes associated with the compounds expected to be identified in human plasma.
- Summed ion intensities.

**Figure 1:**
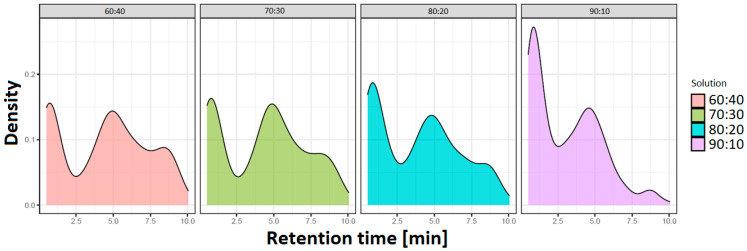
Distribution of ion density over the retention time (between 0.5–10 min) in analyzed plasma extracts prepared using different compositions of acetonitrile: water reconstitution solvent, as indicated.

The PCA score plot of the reconstitution solvent measurements suggested an apparent difference between the groups, as shown by the five distant clusters (Supplementary Material Figure S2). The ion distribution map presented in Figure 1 revealed that the increase in acetonitrile percentage resulted in decreased initial retention and a higher number of ions eluting at the beginning of the chromatogram. This phenomenon can be explained by a more efficient extraction of non-polar compounds that are not retained in a HILIC column by the increased acetonitrile content.

The elution profile of the 90:10 acetonitrile:water extracts was inadequate, while the profiles of the 60:40 and 70:30 groups are considered satisfactory. Due to the described phenomenon, the 90:10 acetonitrile:water group yielded the highest number of detected features, though these did not translate into a higher tentative identification detection rate, as the number of mzCloud matches was similar between all groups, as presented in Table 3. A visual assessment of the peak shape for a panel of common plasma metabolites revealed that the 70:30 group featured the most optimal results. The number of satisfactory peak shapes was the highest among the groups and many features had significantly higher intensity compared to the 80:20 and 90:10 groups. The averaged summed ion intensities (summed intensity of all the peaks in the chromatogram) for each reconstitution group were also investigated. The 70:30 group turned out to have the highest value of this metric, while the group of 90:10 scored the lowest. This may explain the observed number of confident mzCloud matches, as the more intensive signal usually results in a higher quality fragmentation spectrum, which is then a basis for the identification algorithm utilizing mzCloud. Ultimately, the reconstitution solvent composition of 70:30 acetonitrile: water was found to be optimal. In this study, our primary investigation centered on the impact of different acetonitrile–water ratios for sample reconstitution. Notwithstanding, future research could consider evaluating the column equilibration buffer as a potential loading solution to gain additional insights into LC-MS performance.

### 3.2. Injection Volume

The volume of sample extract injected into the LC-MS system can have a significant impact on the peak shape, peak width, and method sensitivity. It, therefore, influences the quality of the data and the number of identified metabolites. In this study, we compared the injection of 1, 2, 3, 4, or 5 µL of sample extract. Assessment of the injection volume effect was based on:

- Total number of features found in MS OT.
- The number of common features in MS OT per group having the highest response (area under the peak) among all five injection volume groups.

The PCA score plot of different injection volume measurements suggested an apparent difference between the groups (Supplementary Material Figure S3). The number of extracted MS OT features were as follows: 656, 830, 859, 906, and 848 in 1, 2, 3, 4, and 5 µL injection volume groups, respectively. The relative standard deviation of the number of filtered MS^1^ features (MS OT) found in the top three groups was 3.5%, suggesting that the values obtained for injection volumes of 3, 4, and 5 µL were relatively similar. 

**Figure 2:**
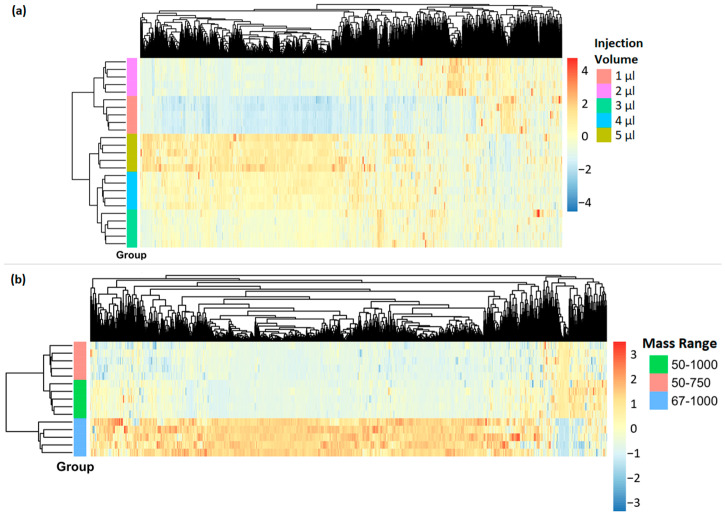
Heatmap analysis representing the intensity of features in plasma extracts: (**a**) injected at different volumes, (**b**) measured using different mass range settings. Each column represents a metabolite feature and each color-coded row represents a sample group.

**Figure 3:**
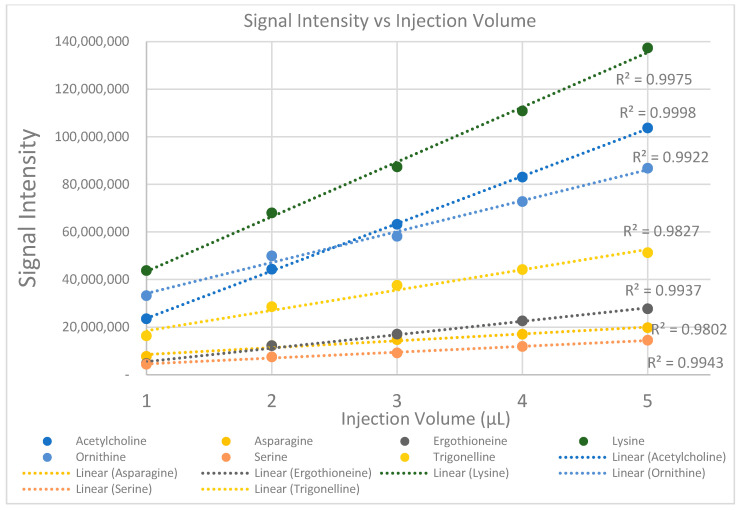
Relationship between injection volume and signal intensity for sample plasma metabolites.

The heatmap, shown in Figure 2a, represents the intensities of particular features in every investigated group. The heatmap revealed that the intensities of the features were evidently the highest in the group of 5 µL. As the injection volume had a more significant effect on the registered intensities compared to the number of detected peaks, we considered the injections of 5 µL to be optimal. No significant ion suppression was observed with an increasing injection volume, which further substantiated the 5 µL group to be the preferred setting. The linearity of response has been assessed on a set of commonly reported plasma metabolites. We have observed a linear relationship between the signal intensity and volume injected for all sample compounds, which is presented in Figure 3.

**Figure 4:**
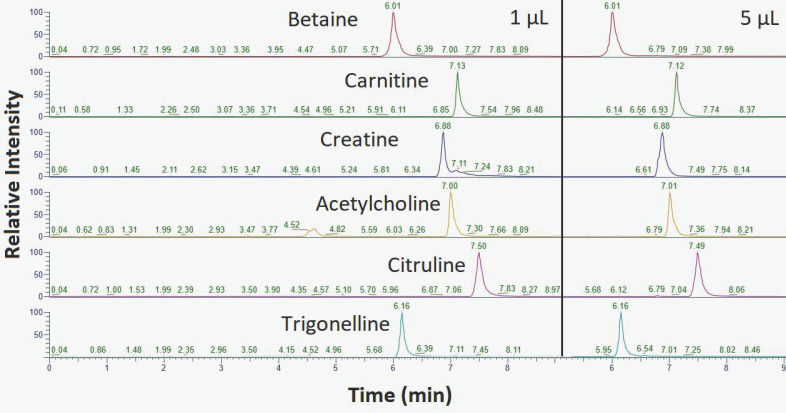
The comparison of the peak shapes for sample compounds acquired with injection volumes set to 1 μL (**left**) and 5 μL (**right**).

The influence of injection volume on a peak shape was also examined visually. The results showed no peak distortion or significant peak broadening by increasing the injection volume, further confirming that the highest injection volume group was the most satisfactory. The comparison of the peak shapes for sample compounds acquired with different injection volumes (1 vs. 5 μL) is presented in Figure 4.

### 3.3. Mass Range

It has been previously reported that the mass range setting of Orbitrap-based mass spectrometers affects the detection rate and sensitivity [23]. Since in untargeted metabolomics small molecules are investigated, we evaluated the following mass ranges: 67–1000 *m*/*z*, which is the range recommended by the manufacturer, as well as 50–750 *m*/*z* and 50–1000 *m*/*z*, to investigate the impact of deviating from the recommended setting. Assessment of this parameter was based on the number of features found in MS OT with the highest area under the peak among the compared groups. We have found that the mass range substantially affected the intensity of registered features, as presented in the heatmap in Figure 2b. This behavior can be linked to the value of the voltage delivered to the focusing lenses (RF) of the C-trap in Orbitrap IQ-X, as the instrument automatically adjusts this voltage to the smallest mass value of the scan range and, once set, it cannot be changed during the measurement. In turn, the mass extraction is optimal for smaller molecules, while for high-mass ions the optimum is not achieved. Thus, it was important to investigate different mass ranges and choose the one providing the highest intensities of measured features, in our case 67–1000 *m*/*z*.

### 3.4. Number of ddMS^2^ OT HCD Scans

The time of a single mass spectrometry experiment cycle is positively correlated with the number of ddMS^2^ OT HCD performed. The Orbitrap IQ-X system allows the user to choose between a fixed number of fragmentation spectra within one cycle or to fix the time of an experiment so that the number of ddMS^2^ OT HCD registered can vary between the experiments.

As we aimed to obtain a uniform number of fragmentation spectra in each cycle, we assessed how the number of MS2 scans—7, 8, 9, 10, and 12—affects the detection and tentative identification rate based on mzCloud. The assessment was based on:

- Total number of features found in MS OT.
- Number of matches with mzCloud Best Match score > 80%.

As presented in Table 4, the group featuring 8 ddMS^2^ OT HCD per cycle was most abundant in MS OT features and second-best in terms of the identifications number, following the group featuring 10 ddMS^2^ OT HCD (101 vs. 104 identifications). Both groups yielded satisfactory results, yet we considered the group with eight scans to be optimal, as the average time of the experiment was shorter (0.95 s and 1.2 s for 8 and 10 ddMS^2^ OT HCD, respectively). Moreover, due to the higher number of MS^1^ and MS^2^ data points generated, this setting would be advantageous for peak quantification and for using a local mass spectral-retention time library in addition to online spectral databases, which is a common strategy employed in untargeted metabolomics.

### 3.5. Collision Energy (CE) Mode

The correct selection of CE has a significant impact on the effective identification of metabolites in MS-based metabolomics. As metabolite profiles vary with different CE, it is a fundamental parameter affecting the quality of fragmentation spectra [24]. In this study, we compared two collision energy modes—stepped and assisted—with different sets of energies applied. An assisted collision energy scan allows for CE optimization during the scan cycle. Small populations of precursor ions are sequentially subjected to increasing collision energies and scanned in the ion trap to determine the optimal CE. Then, only the optimal collision energy is used for the high-resolution ddMS^2^ OT HCD.

With stepped CE, fragments resulting from multiple collision energies are included in a single scan. The precursor ion packet is split into thirds and each packet is fragmented using a different CE so that fragments from all three energies are analyzed in a single scan. Stepped CE increases the likelihood that at least some of the precursor ions experience the optimum CE for fragmentation. The investigated CE modes were as follows (HCD collision energies are expressed in %):

- Stepped CE 10, 20, 40
- Stepped CE 10, 35, 50
- Stepped CE 20, 40, 60
- Assisted CE 10, 20 40
- Assisted CE 20, 35, 50
- Assisted CE 20, 40, 60
- Assisted CE 10, 20, 35, 50
- Assisted CE 10, 20, 35, 50, 60

The assessment was based on:

- The number of features found in MS OT.
- The number of unique MS2 scans.
- The number of matches with mzCloud Best Match score > 80%.

**Figure 5:**
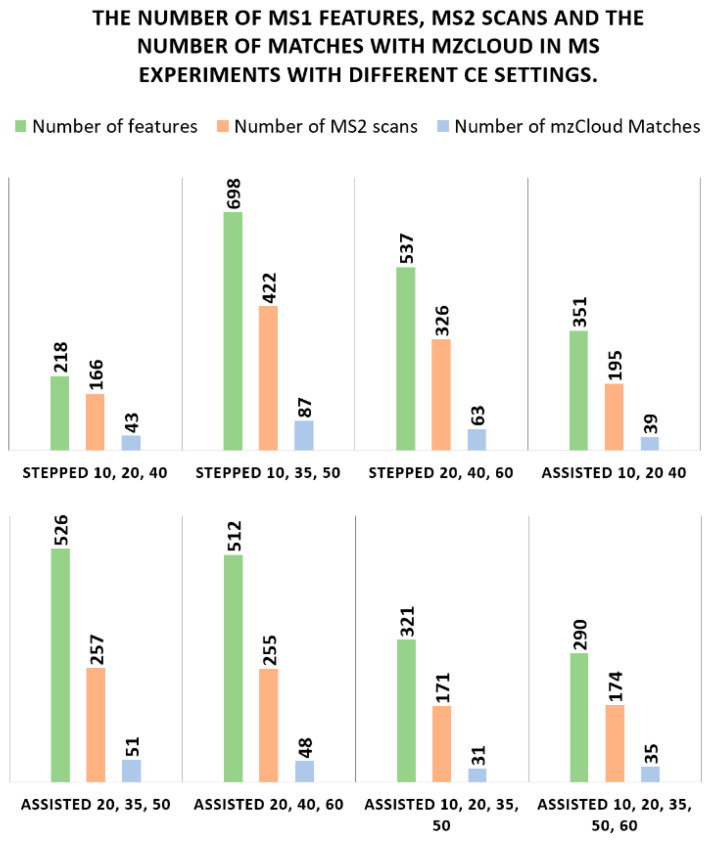
Number of features detected in MS OT, number of unique MS^2^ scans, number of matches with mzCloud Best Match score > 80%, and the number of features displaying the highest intensities among all the groups in MS experiments using different resolution settings.

We have observed an increased number of fragments and fragmentation spectra recorded over the peak in stepped CE mode when compared to the assisted CE mode. MS^2^ spectra acquired with stepped energy had better quality with more fragment ions than spectra acquired in assisted CE mode, which translated into a higher number of mzCloud matches. Moreover, stepped CE featured a three to five-fold increase in MS^1^ points per peak compared to assisted CE. Eventually, stepped CE 10, 35, 50 turned out to be the most optimal, yielding the highest number in all investigated parameters, as presented in Figure 5. The optimal CE covered approximately 95% of mzCloud matches found in other experiments while revealing a few novel matches.

### 3.6. Dynamic Exclusion Time

Dynamic exclusion is a computational method minimizing the repeat selection of identical precursor ions for HCD fragmentation in subsequent mass spectrometry experiment cycles [25]. To increase metabolome coverage, the selected mass is temporarily added to the dynamic exclusion list for a selected period of time, allowing the instrument to fragment other less abundant ions. This permits the mass spectrometer to analyze more compounds instead of repeatedly sequencing the same abundant metabolites [26]. Setting the duration of dynamic exclusion is particularly challenging, as in UPLC-MS some peaks widths can be very small, even 1–2 s wide. In our method, each precursor was added to the exclusion list after a single detection in the MS cycle with a low and high mass tolerance of 5 ppm. We compared exclusion times of: 1.5, 2.5, 3.5, and 5 s and assessed them by the number of unique features subjected to fragmentation (regardless if features were annotated or not), which were 456, 512, 351, and 59, respectively. A dynamic exclusion time of 2.5 s was found to be optimal, resulting in the highest number of unique MS^2^ spectra.

### 3.7. Mass Resolution of Scan Event

In a mass spectrum, resolution can be defined as the observed *m*/*z* value divided by the smallest difference Δ(*m*/*z*) for two ions that can be separated: (*m*/*z*)/Δ(*m*/*z*) [27]. Mass resolution is typically a large number that describes the ability to distinguish between ions differing in the *m*/*z* value by a small increment. The mass resolution is characterized by the peak width, measured in mass units, expressed as a function of the mass, for at least two points on the peak. In mass spectrometers where Orbitrap technology is employed, this is reported at 50% of the maximum peak height (Full Width at Half Maximum—FWHM). As we aimed to obtain high-resolution data for both scans in the mass spectrometry experiments, MS OT and ddMS^2^ OT HCD, these were measured in the Orbitrap. We compared a set of three different MS OT resolutions: 50,000, 60,000, and 120,000 in addition to two ddMS^2^ OT HCD settings: 15,000 and 30,000.

The resolution setting reported by the manufacturer of Orbitrap IQ-X corresponds to the measurement at *m*/*z* 200, as in Orbitrap-based instruments the resolution is inversely proportional to the *m*/*z* (it decreases with an increase of ion mass) [28,29]. Spectra acquired at a higher Orbitrap resolution allow higher resolution of the mass peaks; however, they take a longer time to acquire. The assessment was based on:

- Number of features found in MS OT.
- Number of unique MS2 scans.
- Number of matches with mzCloud Best Match score > 80%.

The results are presented in Table 5. The resolution setting showed a varying impact on the number of detected compounds, fragmentation spectra, and mzCloud matches.

The group featuring a resolution of 120 k in MS OT and 15 k in ddMS^2^ OT HCD was found to be optimal, as it yielded the highest number of features measured, provided an optimal balance between MS^1^ and MS^2^ scans per peak, and featured the highest number of unique fragmentation spectra. The relative standard deviation of the number of mzCloud matches between groups was below 5%. Therefore, this parameter was not considered a major factor influencing the quality of the results.

## 4. Conclusions

In this study, human plasma extract was used to investigate the influence of reconstitution solvent mixture and LC-MS instrument settings on the quality of collected spectral data and the effect on tentative identification with an online spectral library. As demonstrated, these considerations and operating parameters of the Orbitrap IQ-X system are essential for reliable data collection and significantly influence the outcome of data analysis. Using UPLC–MS/MS as a technique of choice, we showed that different MS conditions can notably affect metabolomics data output. For example, the selected mass range has a significant impact on the sensitivity of the instrument. Based on our extensive studies, we presented recommendations for reconstitution solvent composition and injection volume for plasma extracts prepared using the given sample preparation protocol, and five relevant MS and MS/MS settings as summarized in Table 6, that resulted in an approximately 40% increase of reported mzCloud matches. Such a methodological framework for method optimization is applicable to various cutting-edge MS systems including Orbitrap Eclipse™ Tribrid™, Orbitrap Ascend Tribrid™, and Orbitrap Fusion™ Lumos™ Tribrid™, among others. We hope that this work will provide a useful approach to understanding and optimizing vital parameters for untargeted metabolomics studies of biological samples.

## Figures and Tables

**Table 1:** Mobile phase gradient of LC-MS method.

Time (min)	Mobile Phase Composition (%)	
	A	B
Initial	99	1
1	99	1
3	85	15
6	50	50
9	5	95
10	5	95
10.5	99	1
15	99	1

**Table 2:** Base parameters of the metabolomics LC-MS method.

Parameter	Value
Reconstitution Solvent, Acetonitrile/Water Mass Range	80:20 (*v*/*v*)
	67–1000 *m*/*z*
RF Lens Amplitude	35%
Injection Volume	5 µL
Number of ddMS^2^ OT HCD Scans per Cycle	8
HCD Collision Energy Mode (normalized)	Stepped 20, 40, 60%
Dynamic Exclusion Time	2.5 s
MS OT Resolution	60 k
Intensity Threshold	2.0 e4
ddMS^2^ Resolution	30 k
MS OT Normalized Automatic Gain Control (AGC) Target	100%
MS OT Maximum Injection Time (custom)	50 ms
ddMS^2^ OT Normalized AGC Target	50%
Maximum Injection Time (custom)	54 ms

**Table 3:** The number of unique MS^1^ features, mzCloud matches with over 80% best match score, and averaged summed ion intensities in LC-MS experiments using different reconstitution solvent compositions during sample preparation.

Reconstitution Solvent Composition (Acetonitrile:Water)	Number of Features (MS OT)	Number of mzCloud Matches	Summed Ion Intensities
60:40	781	97	1.13 E + 10
70:30	736	105	1.37 E + 10
80:20	795	103	1.14 E + 10
90:10	872	86	9.97 E + 10

**Table 4:** The number of features detected in MS OT and matches with mzCloud featuring Best Match score >80% in experiments employing a different number of fragmentation events per cycle.

Number of ddMS^2^ OT HCD Per Cycle	Number of Features (MS OT)	Number of mzCloud Matches
7	732	90
8	755	101
9	713	100
10	735	104
12	621	93

**Table 5:** Number of features detected in MS OT, number of unique MS^2^ scans, number of matches with mzCloud Best Match score > 80%, and the number of features displaying the highest intensities among all the groups in MS experiments using different resolution settings.

Resolution		Number of Features	Number of Unique MS^2^	Number of mzCloud Matches
Full MS	MS^2^			
120 k	30 k	840	466	105
120 k	15 k	896	509	103
60 k	30 k	775	445	99
60 k	15 k	808	506	107
50 k	30 k	723	445	93
50 k	15 k	744	504	101

**Table 6:** Optimal parameters identified for the metabolomics LC-MS method.

Parameter	Value
Reconstitution solvent composition, Acetonitrile/Water	70:30 (*v*/*v*)
Injection Volume	5 µL
Mass range	67–1000 *m*/*z*
Number of ddMS^2^ OT HCD scans per cycle	8
HCD Collision Energy Mode (normalized)	Stepped 10, 35, 50%
Dynamic Exclusion Time	2.5
MS OT Resolution	120 k
ddMS^2^ Resolution	15 k

## Data Availability

Research data may be provided upon reasonable request. Data is not publicly available due to privacy.

## Supplementary Materials

The following supporting information can be downloaded at: https://www.mdpi.com/article/10.3390/metabo13080923/s1, Figure S1: Untargeted Metabolomics Data Analysis Workflow in Compound Discoverer; Figure S2: Principle component analysis (PCA) score plot—first and second principal components of plasma sample extracts prepared using different acetonitrile: water reconstitution solvents, as indicated.; Figure S3: Principle component analysis (PCA) score plot—first and second principal components of plasma sample extracts injected at different volumes. Figure S4: PCA score plot - first and second principal components of plasma sample extracts prepared using different mass range settings.

## References

[1] Patti G.J., Yanes O., Siuzdak G. (2012). Metabolomics: The apogee of the omics trilogy. Nat. Rev. Mol. Cell Biol..

[2] Roca M., Alcoriza M.I., Garcia-Cañaveras J.C., Lahoz A. (2021). Reviewing the metabolome coverage provided by LC-MS: Focus on sample preparation and chromatography-A tutorial. Anal. Chim. Acta.

[3] Roberts L.D., Souza A.L., Gerszten R.E., Clish C.B. (2012). Targeted Metabolomics. Curr. Protoc. Mol. Biol..

[4] Vinayavekhin N., Saghatelian A. (2010). Untargeted Metabolomics. Current Protocols in Molecular Biology.

[5] Lelli V., Belardo A., Timperio A.M. (2021). From Targeted Quantification to Untargeted Metabolomics. Metabolomics—Methodology and Applications in Medical Sciences and Life Sciences.

[6] Zhang X., Zhu X., Wang C., Zhang H., Cai Z. (2016). Non-targeted and targeted metabolomics approaches to diagnosing lung cancer and predicting patient prognosis. Oncotarget.

[7] Vaniya A., Fiehn O. (2015). Using fragmentation trees and mass spectral trees for identifying unknown compounds in metabolomics. TrAC Trends Anal. Chem..

[8] Williamson J.C., Edwards A.V.G., Verano-Braga T., Schwämmle V., Kjeldsen F., Jensen O.N., Larsen M.R. (2016). High-performance hybrid Orbitrap mass spectrometers for quantitative proteome analysis: Observations and implications. Proteomics.

[9] Shah S.H., Kraus W.E., Newgard C.B. (2012). Metabolomic Profiling for the Identification of Novel Biomarkers and Mechanisms Related to Common Cardiovascular Diseases. Circulation.

[10] Cubbon S., Antonio C., Wilson J., Thomas-Oates J. (2010). Metabolomic applications of HILIC-LC-MS. Mass Spectrom. Rev..

[11] Tang D.-Q., Zou L., Yin X.-X., Ong C.N. (2016). HILIC-MS for metabolomics: An attractive and complementary approach to RPLC-MS. Mass Spectrom. Rev..

[12] Morán-Garrido M., Muñoz-Escudero P., García-Álvarez A., García-Lunar I., Barbas C., Sáiz J. (2022). Optimization of sample extraction and injection-related parameters in HILIC performance for polar metabolite analysis. Application to the study of a model of pulmonary hypertension. J. Chromatogr. A.

[13] Spagou K., Tsoukali H., Raikos N., Gika H., Wilson I.D., Theodoridis G. (2010). Hydrophilic interaction chromatography coupled to MS for metabonomic/metabolomic studies. J. Sep. Sci..

[14] Medina J., Van Der Velpen V., Teav T., Guitton Y., Gallart-Ayala H., Ivanisevic J. (2020). Single-Step Extraction Coupled with Targeted HILIC-MS/MS Approach for Comprehensive Analysis of Human Plasma Lipidome and Polar Metabolome. Metabolites.

[15] Trivedi D.K., Iles R.K. (2015). HILIC-MS-based shotgun metabolomic profiling of maternal urine at 9–23 weeks of gestation—Establishing the baseline changes in the maternal metabolome. Biomed. Chromatogr..

[16] Røst L.M., Shafaei A., Fuchino K., Bruheim P. (2020). Zwitterionic HILIC tandem mass spectrometry with isotope dilution for rapid, sensitive and robust quantification of pyridine nucleotides in biological extracts. J. Chromatogr. B.

[17] Virgiliou C., Sampsonidis I., Gika H.G., Raikos N., Theodoridis G.A. (2015). Development and validation of a HILIC-MS/MS multitargeted method for metabolomics applications. Electrophoresis.

[18] Ivanisevic J., Zhu Z.-J., Plate L., Tautenhahn R., Chen S., O’brien P.J., Johnson C.H., Marletta M.A., Patti G.J., Siuzdak G. (2013). Toward ‘Omic Scale Metabolite Profiling: A Dual Separation–Mass Spectrometry Approach for Coverage of Lipid and Central Carbon Metabolism. Anal. Chem..

[19] Periat A., Boccard J., Veuthey J.-L., Rudaz S., Guillarme D. (2013). Systematic comparison of sensitivity between hydrophilic interaction liquid chromatography and reversed phase liquid chromatography coupled with mass spectrometry. J. Chromatogr. A.

[20] Southam A.D., Haglington L.D., Najdekr L., Jankevics A., Weber R.J.M., Dunn W.B. (2020). Assessment of human plasma and urine sample preparation for reproducible and high-throughput UHPLC-MS clinical metabolic phenotyping. Analyst.

[21] Reed G.F., Lynn F., Meade B.D. (2002). Use of Coefficient of Variation in Assessing Variability of Quantitative Assays. Clin. Vaccine Immunol..

[22] Li H., Liu C., Zhao L., Xu D., Zhang T., Wang Q., Cabooter D., Jiang Z. (2021). A systematic investigation of the effect of sample solvent on peak shape in nano- and microflow hydrophilic interaction liquid chromatography columns. J. Chromatogr. A.

[23] Fall F., Lenuzza N., Lamy E., Brollo M., Naline E., Devillier P., Thévenot E., Grassin-Delyle S. (2019). A split-range acquisition method for the non-targeted metabolomic profiling of human plasma with hydrophilic interaction chromatography—High-resolution mass spectrometry. J. Chromatogr. B.

[24] Madala N.E., Steenkamp P.A., Piater L.A., Dubery I.A. (2012). Collision energy alteration during mass spectrometric acquisition is essential to ensure unbiased metabolomic analysis. Anal. Bioanal. Chem..

[25] Kohli B.M., Eng J.K., Nitsch R.M., Konietzko U. (2005). An alternative sampling algorithm for use in liquid chromatography/tandem mass spectrometry experiments. Rapid Commun. Mass Spectrom..

[26] Hodge K., Have S.T., Hutton L., Lamond A.I. (2013). Cleaning up the masses: Exclusion lists to reduce contamination with HPLC-MS/MS. J. Proteom..

[27] Murray K.K., Boyd R.K., Eberlin M.N., Langley G.J., Li L., Naito Y. (2013). Definitions of terms relating to mass spectrometry (IUPAC Recommendations 2013). Pure Appl. Chem..

[28] Makarov A., Denisov E., Kholomeev A., Balschun W., Lange O., Strupat K., Horning S. (2006). Performance Evaluation of a Hybrid Linear Ion Trap/Orbitrap Mass Spectrometer. Anal. Chem..

[29] Hardman M., Makarov A.A. (2003). Interfacing the Orbitrap Mass Analyzer to an Electrospray Ion Source. Anal. Chem..

