# Supplementary material for: Optimization Strategies for Mass Spectrometry-Based Untargeted Metabolomics Analysis of Small Polar Molecules in Human Plasma
Source: Metabolites. 2023 Aug 7;13(8):923. doi: 10.3390/metabo13080923 (PMC10456887; doi:10.3390/metabo13080923)
Supplement: Supplementary file 1 [file metabolites-13-00923-s001.zip › metabolites-2496577-supplementary.pdf]

Supplementary Materials

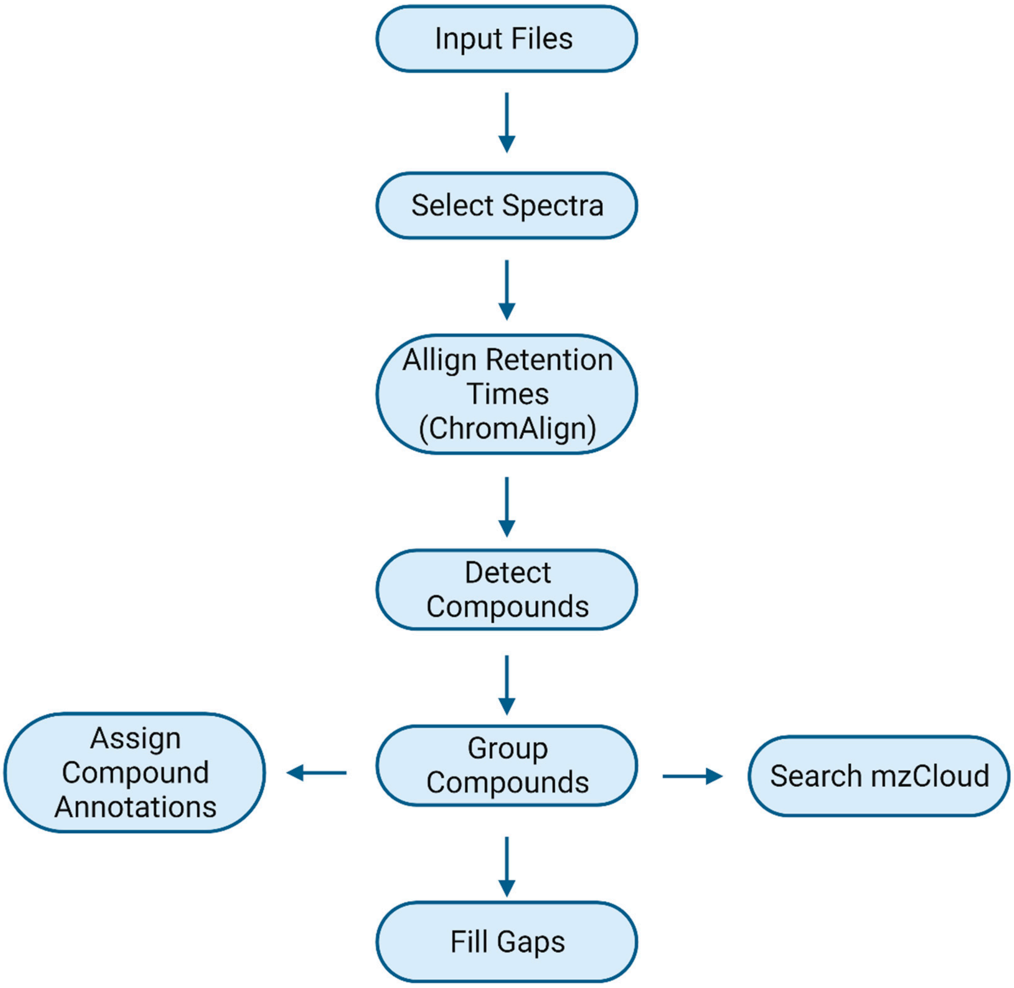

Figure S1. Untargeted Metabolomics Data Analysis Workflow in Compound Discoverer.

During the data processing according to the presented workflow, the following processes take place:

I. The Input Files node sends the file names and location of the input files to the Select Spectra node.

II. The Select Spectra node reads the .RAW data files and filters the mass spectral scan data.

III. The Align Retention Times (ChromAlign) node chromatographically aligns features across the input files in a sample set by using the specified alignment algorithm. The node finds features that are common across most samples and builds correlation matrices based on spectral similarities. Then the node builds regression curves by using the optimal path in the correlation matrix. The node uses a reference file to build these matrices.

IV. The Detect Compounds node detects contiguous mass traces in the full (MS1) scans by using the parameter settings for the mass tolerance and intensity threshold and detects chromatographic peaks in the contiguous mass traces. The node uses the peak quality information to group the extracted-ion chromatogram (XIC) traces for common isotopes of C, H, N, O, and S (optionally for Cl and Br). It includes peak quality thresholds for filtering out XIC traces with low-quality chromatographic peaks for isotope grouping. The Detect Compounds node also groups adducts by using user-specified ions and base ions lists.

V. The Group Compounds node uses the specified mass and RT tolerances to group chromatographic peaks with the same molecular weight (MW) × RT values. The node also filters out compounds that do not pass the peak quality threshold in the specified number of input files. It then sends the best fragmentation data across the input files to the Search mzCloud node and Predict Compositions node.

This node also selects the best hit ions for each compound across the input file set:

- Selects the best ion and related MS^1 scan for each compound as the one with the highest resolution and the highest intensity for the preferred ion.

- Selects the best fragmentation data by using the user-specified preferred precursor ion with the highest intensity that has data-dependent MS^2 scans.

VI. The Search mzCloud node searches the mzCloud database (www.mzcloud.org) for matching and similar fragmentation spectra.

VII. The Predict Compositions node predicts the elemental compositions of the unknown compounds.

VIII. The Fill Gaps node fills in missing peaks or peaks below the detection threshold (specified in the Detect Compounds node) for subsequent statistical analysis.

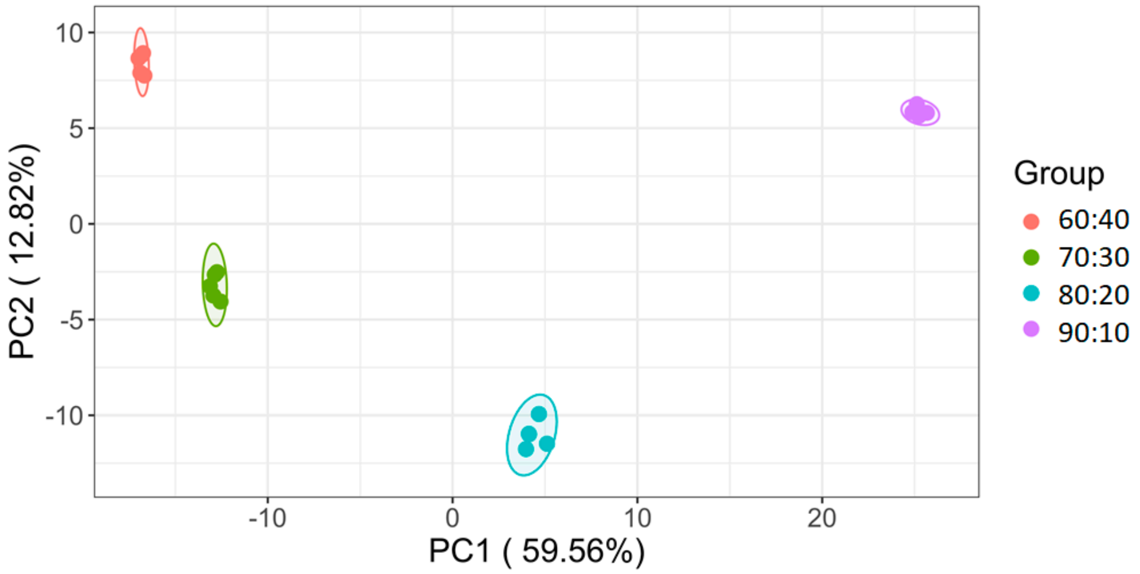

Figure S2. Principle component analysis (PCA) score plot - first and second principal components of plasma sample extracts prepared using different acetonitrile: water reconstitution solvents, as indicated.

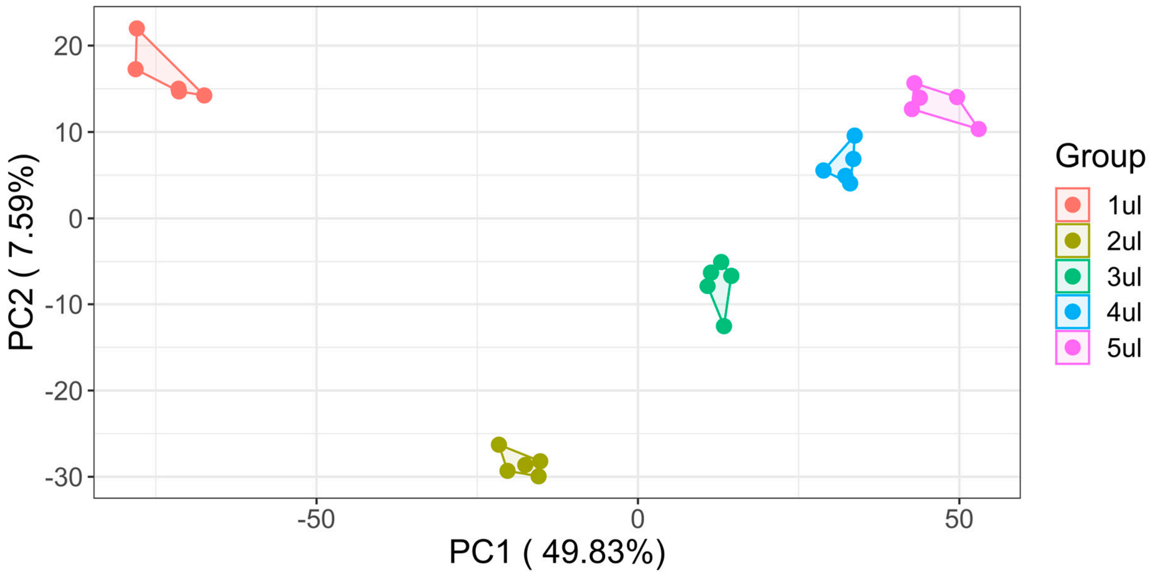

Figure S3. Principle component analysis (PCA) score plot - first and second principal components of plasma sample extracts injected at different volumes.

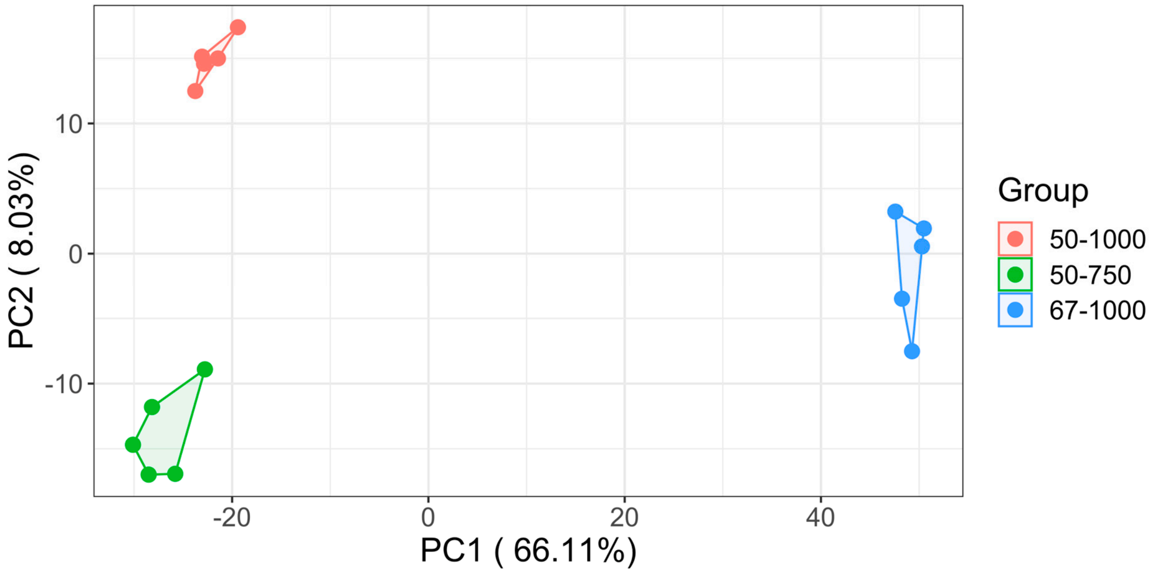

Figure S4. PCA score plot - first and second principal components of plasma sample extracts prepared using different mass range settings.
